# Parental LTRs Are Important in a Construct of a Stable and Efficient Replication-Competent Infectious Molecular Clone of HIV-1 CRF08_BC

**DOI:** 10.1371/journal.pone.0031233

**Published:** 2012-02-17

**Authors:** Qiwei Zhang, Xiaomin Zhang, Hao Wu, Donald Seto, Hao-Jie Zhang, Zhiwei Chen, Chengsong Wan, Bo-Jian Zheng

**Affiliations:** 1 Department of Microbiology, Li Ka Shing Faculty of Medicine, The University of Hong Kong, Hong Kong SAR, China; 2 Department of Microbiology, School of Public Health and Tropical Medicine, Southern Medical University, Guangzhou, China; 3 Bioinformatics and Computational Biology, School of Systems Biology, George Mason University, Manassas, Virginia, United States of America; 4 AIDS Institute, Li Ka Shing Faculty of Medicine, The University of Hong Kong, Hong Kong SAR, China; Johns Hopkins School of Public Health, United States of America

## Abstract

Circulating recombinant forms (CRFs) of HIV-1 have been identified in southern China in recent years. CRF08_BC is one of the most predominant subtypes circulating in China. In order to study HIV subtype biology and to provide a tool for biotechnological applications, the first full-length replication-competent infectious molecular clone harboring CRF08_BC is reported. The construction of this clone pBRGX indicates that a moderate-copy number vector is required for its amplification in *E. coli*. In addition, it is shown that the parental CRF08_BC LTRs are important for generating this efficient replication-competent infectious clone. These observations may aid in the construction of infectious clones from other subtypes. Both the pBRGX-derived virus and its parental isolate contain CCR5 tropism. Their full-length genomes were also sequenced, analyzed, compared and deposited in GenBank (JF719819 and JF719818, respectively). The availability of pBRGX as the first replication-competent molecular clone of CRF08_BC provides a useful tool for a wide range of studies of this newly emergent HIV subtype, including the development of HIV vaccine candidates, antiviral drug screening and drug resistance analysis.

## Introduction

Since the first report of an HIV-1 infection in Beijing in 1985 [1], HIV and its resultant disease, AIDS, have spread rapidly throughout the country and have become a major concern in China. The local epidemics of HIV-1 subtype B′ (Thai-B) in 1989 and subtype C in 1992, among intravenous drug users (IDUs) in Yunnan, triggered the explosive nation-wide HIV-1 epidemic in China [2], [3]. By the end of the year 2010, China had recorded 379,348 cases of HIV infections, including 138,288 cases that progressed to full-blown AIDS with 72,616 fatalities [4]. The Chinese Center for Disease Control and Prevention (CDC) recently estimated that there may be as many as 740,000 HIV-infected individuals at the present, almost twice the reported number [4]. The Circulating Recombinant Form (CRF) CRF08_BC presumably originated among IDUs in Yunnan, which is a major entry point for heroin smuggling into China, and then likely spread along known drug trafficking routes to other districts, such as the Guangxi and Xinjiang regions of China in 1997 [5]–[7]. In 2010, Shang *et al*., surveyed HIV-1 positive, “injection drug users”, “former paid blood donors (FBD)” and sexually-transmitted disease cases from multiple provinces across China to assess subtypes [7]. The current circulating subtypes prevalent are CRF07_BC, CRF08_BC, B′ and CRF01_AE; the CRF08_BC strain has become one of the most predominant circulating HIV-1 strains among these subtypes [7]–[11]. For example, the Guangxi region, east Yunnan province (near Guangxi) and Liaoning province (northeastern China) have become three of the most heavily impacted populations in China due to this particular subtype [12]–[14].

Genome recombination drives the molecular evolution of HIV and results in the large genetic diversity found amongst HIV-1 isolates. This molecular mechanism generates new mosaic strains frequently, particularly in populations where multiple subtypes co-circulate. Replication-competent infectious molecular clones are required for, and have been widely used for, assessing potential biotechnological solutions to the HIV problem. These include the evaluation of the immunogenicity of HIV vaccines; screens of prospective antiviral drugs; and analysis of antiviral drug resistance. To date, infectious molecular clones from HIV-1 subtypes A, B, B′, C, D and O, as well as CRFs A/E, A/G, D/C and 07_BC, have been constructed [9], [10], [15]–[29]. For subtype CRF08_BC, however, none has been reported, although it is the strain that is predominantly circulating in China currently. As the foundation for additional and more precise characterization of this strain, and for the support of efforts to manipulate genetically HIV-1 CRF08_BC, e.g., vaccine development, a replication-competent infectious molecular clone containing the full proviral genome of the strain isolated from the Guangxi region of China was constructed using pBR322, a moderate-copy number plasmid. Two non-infectious molecular clones harboring most of the proviral genome, but missing their parental LTRs, were also generated; these were compared to the infectious molecular clone. All three clones were characterized, including the determination of full-length genome sequences from pBRGX and its parental isolate 2007CNGX-HK. One important observation is the apparent need for the inclusion of the parental LTRs in order to propagate, recover and maintain the intact, full-length infectious molecular clone; another is that a very high-copy number plasmid, such as pUC, is not as optimal as a moderate-copy number plasmid, e.g., pBR322, at least for subtype CRF08_BC. Both observations have implications for future work in generating additional infectious molecular clones of HIV and possibly other similar viruses.

## Materials and Methods

### Virus origin, isolation and growth

HIV-1 primary virus strain 2007CNGX-HK was provided by one of the authors (ZC). It was isolated at the University of Hong Kong AIDS (HKU-AIDS) Institute in 2007 from an intravenous drug user in the Guangxi region of southern China, who was symptomatic and diagnosed in 2007. Peripheral blood mononuclear cells (PBMCs) from the virus cultures were separated by Ficoll-Hypaque density gradient centrifugation, according to the manufacturer's protocol. For virus isolation, PBMCs were co-cultured with phytohemagglutinin (PHA) (5 µg/ml)-stimulated PBMCs from HIV-negative healthy donors in RPMI 1640 medium containing fetal calf serum (10%), interleukin-2 (20 ng/ml) and polybrene (5 µg/ml) for 30 days. HIV-infected PBMCs were harvested and proviral DNA was purified using QIAamp DNA Blood Mini Kits (QIAGEN Pte. Co., Ltd; Hong Kong), according to the manufacturer's instructions. This resultant DNA was suitable for genome sequencing and for use as polymerase chain reaction (PCR) templates.

### Construction of an infectious molecular clone from 2007CNGX-HK

PCR using AccuPrime™ Taq DNA Polymerase High Fidelity (Invitrogen) provided near-full-length proviral DNA using the following primers: *Bss*HF (5′-GCTGAAGCGCGCTCGGCAAGAGGCGAGA-3′; the *Bss*HII site underlined) and XhoR (5′-CCAGGTCTCGAGATACTGCTCCCACCCCATCTGC-3′; the *Xho*RI site underlined). It is noted as “near-full-length” since the genome was missing its homologous LTRs, i.e., the left and right ends of the genome. After *Bss*HII and *Xho*I digestion, the eight kilobase (kb) PCR product (containing all of the open reading frames except Nef) was cloned into a pCR-XL-TOPO TA vector (Invitrogen Corp.; USA). A positive clone was screened, recovered and subcloned into pNL4-3, a previous described replication-competent clone of subtype B [15] (a generous gift from the NIH AIDS Research and Reference Reagent Program; National Institute of Allergy and Infectious Diseases (NIAID), National Institutes of Health (NIH); Bethesda, MD) to generate pNGX5. pNGX5 is a chimeric plasmid harboring the majority of the genome of 2007CNGX-HK, i.e., missing its LTRs, with the resident 5′ and 3′ LTRs derived from pNL4-3. This clone was rescued by subcloning into a moderate-copy number plasmid, pBR322, after the *Aat*II and *Ngo*MIV digestion of both plasmids, resulting in pBRNG. Finally, pBRGX was generated by exchanging the pBRNG LTRs with the parental LTRs from 2007CNGX-HK. For this LTR exchange, briefly, the 5′ LTR was PCR-amplified using primers GXU3AF (5′-CGCGACGTCTGGAAGGGTTAATTTACTCTAAGA-3′; the *Aat*II site underlined) and GXgagSR (5′-CTGAAGAGTACTAGTAGTTCCTGCTATGTC-3′; the *Spe*I site underlined) while the 3′ LTR was amplified using primers GX-8860X-F (5′-GGGAGCAGTATCTCGAGACCTGGA-3′; the *Xho*I site underlined) and GXU5NR (5′-ATAAGCCGGCTGCTAGAGATTTTCCACACTACCAC-3′; the *Ngo*MIV site underlined). The final plasmid pBRGX construct contains the full-length DNA version of the 2007CNGX-HK genome including its homologous LTRs.

### Transfection and viral replication kinetics

For the recovery of infectious virion, the molecular clones pBRNG and pBRGX were transfected into 293FT cells using Lipofectamine 2000 (Invitrogen Corp.; USA). The p24 antigen level, indicative of HIV gene expression and viral replication, in the culture supernatant, was detected at 72 h post-transfection by an ELISA assay (bioMérieux; France), performed as described previously [30]. Viral infectivity in TZM-bl cells was determined by an X-Gal Staining assay, as described previously [31], [32]. Replication kinetics of the viruses from the supernatants recovered from both the pBRNG- and pBRGX-transfected 293FT cells were tested further in PBMC cells. Briefly, this called for 4×10^6^ donor PBMCs, stimulated with IL-2/PHA, to be inoculated with supernatant (1 ml) from transfected 293FT cells that were normalized at 40 ng p24 (for pBRNG, 1 ml virus stock was used for infection). At two hours post-infection, the cells were washed twice with PBS and cultured at 37°C in 2 ml fresh medium. Cultures were tested for the presence of the p24 antigen in the supernatant every 3–4 days, and fed with 1 ml fresh PHA-stimulated and polybrene-treated PBMCs (2×10^6^ cells) every 7 days. The parental isolate 2007CNGX-HK strain, normalized at 40 ng p24, was also cultured in PBMCs as a positive control.

### Co-receptors mediated assay

The co-receptor tropism of the infectious clone pBRGX and its parental isolate was identified using U373-MAGI-CXCR4cem, U373-MAGI-CCR5e and U373MAGI cells. These cells were obtained from the NIH AIDS Research and Reference Reagent Program. Briefly, 0.6×10^5^ cells were dispensed to each well in a 96-well microplate. These were infected with 50 µl of serially-diluted virus from the pBRGX transfection supernatant, in parallel experiments with its parental isolate, and incubated for 2 h. Fresh medium was added and the cells were cultured for 48 h at 37°C. The viral infection was scored as “positive” if it resulted in the blue staining of the infected cell nuclei upon the addition of the beta-galactosidase substrate “X-Gal” (5-bromo-4-chloro-3-indolyl-b-D-galactopyranoside) [32], [33]. The assay was performed in triplicate for reproducibility.

### Sequencing and phylogenetic analysis

The proviral genomes of both the parental isolate and the infectious clone pBRGX were sequenced using a primer-walking protocol and the Sanger dideoxynucleotide chemistry, with three-fold coverage and complementary reads. These sequencing ladders were assembled with the Seqman Pro software, as described previously [34]. Nucleotide and amino acid sequences were aligned with CLUSTAL and BLAST software. These whole genome sequences were annotated and deposited into GenBank (accession numbers JF719819 and JF719818, respectively).

Genome sequence recombination analysis was performed using the Recombinant Identification Program (RIP 3.0), available from the Los Alamos HIV sequence database (http://www.hiv.lanl.gov/content/sequence/RIP/RIP.html). Phylogenetic analysis and trees were generated using a neighbor-joining method implemented in MEGA 5 (Molecular Evolutionary Genetics Analysis) [35], [36] with 1000 bootstrap replicates. The evolutionary distances were computed using the Maximum Composite Likelihood method [37]. Reference HIV-1 genome sequences of subtypes B, C, 07BC and 08BC were obtained from the Los Alamos HIV sequence database (http://www.hiv.lanl.gov/content/index).

### Statistical analysis

The data were evaluated for statistical significance using the Student's *t*-test. Values of *P*<0.05 were considered significant. Results were given as the mean ± standard deviation (SD) of triplicate independent experiments.

## Results

### Construction of the full-length molecular clones pNGX5, pBRNG and pBRGX

**Figure 1:**
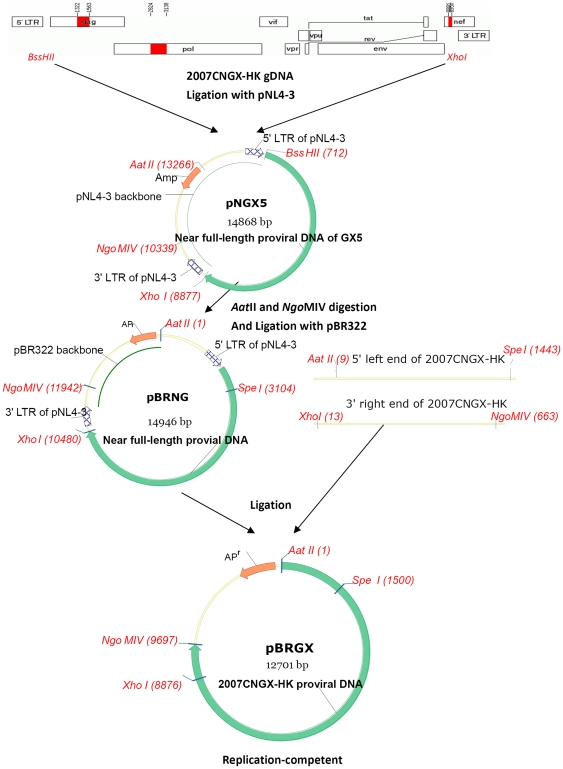
Construction of HIV-1 CRF08_BC infectious clone. Proviral DNA from 2007CNGX-HK, without its homologous LTRs, was PCR-amplified and subcloned into pNL4-3. This resulted in the chimeric clone pNGX5 harboring a majority of the genome of 2007CNGX-HK, that is, except for the homologous LTRs as indicated. Subsequently, the full-length genome of pNGX5 was subcloned in pBR322 after *Aat*II and *Ngo*MIV digestion. The 5′ LTR and 3′ LTR of the resultant plasmid pBRNG were then exchanged with those from 2007CNGX-HK, using *Aat*II and *Spe*I restriction digestion for the 5′ LTR and using *Ngo*MIV and *Xho*I restriction digestion for the 3′ LTR. The final construct pBRGX was 12,701 bp and was replication-competent.

The proviral DNA from HIV-1 CRF_08BC strain 2007CNGX-HK was extracted from infected PBMCs. As shown in Fig. 1, near-full-length proviral DNA, i.e., missing its homologous LTRs, was amplified by PCR and inserted into the pNL4-3 vector, a pUC-based vector, which resulted in a chimeric vector pNGX5 with both LTRs originating from pNL4-3. However, this construct was found to be very unstable in *E. coli* hosts upon culturing (strains DH5α and JM109). It was observed that after two passages, the plasmid sizes decreased, presumably due to deletion and/or recombination. Therefore, the full viral genome, harboring both LTRs from pNL4-3, was subcloned into pBR322, a moderate-copy number vector, in order to generate pBRNG. As the pBRNG construct contains LTRs from pNL4-3 rather than from the primary isolate, these were exchanged with the ones from 2007CNGX-HK, resulting in clone pBRGX. This clone contained the full-length proviral DNA, including the parental LTRs from subtype 2007CNGX-HK. Unlike pNGX5, both pBRNG and pBRGX constructs were stable in *E. coli* cultures, including strains DH5α and JM109, even after 20 passages of cultures.

### Parental LTRs are important for an efficient replication-competent infectious molecular clone

**Figure 2:**
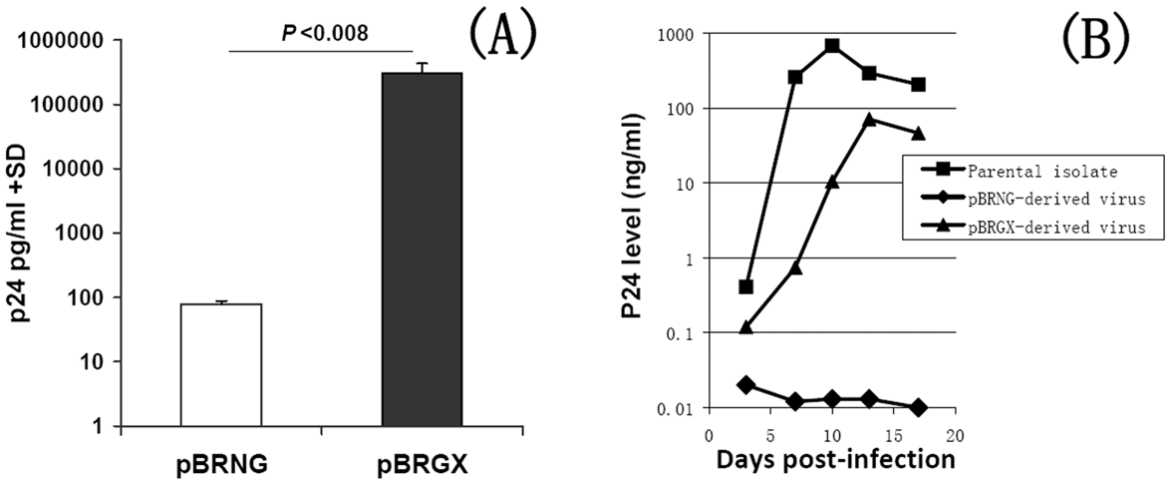
Replication kinetics of HIV-1 CRF_08BC molecular clones pBRNG and pBRGX and its parental isolate. (A) The p24 levels in the supernatants from pBRNG and pBRGX-transfected 293FT cells are shown. (B) The p24 levels in the PBMCs infected with pBRNG and pBRGX-derived virus, as well as the parental isolate are noted at days 3, 7, 10, 13 and 17 post-infection. An ELISA assay was used to assay the p24 antigen concentrations.

**Figure 3:**
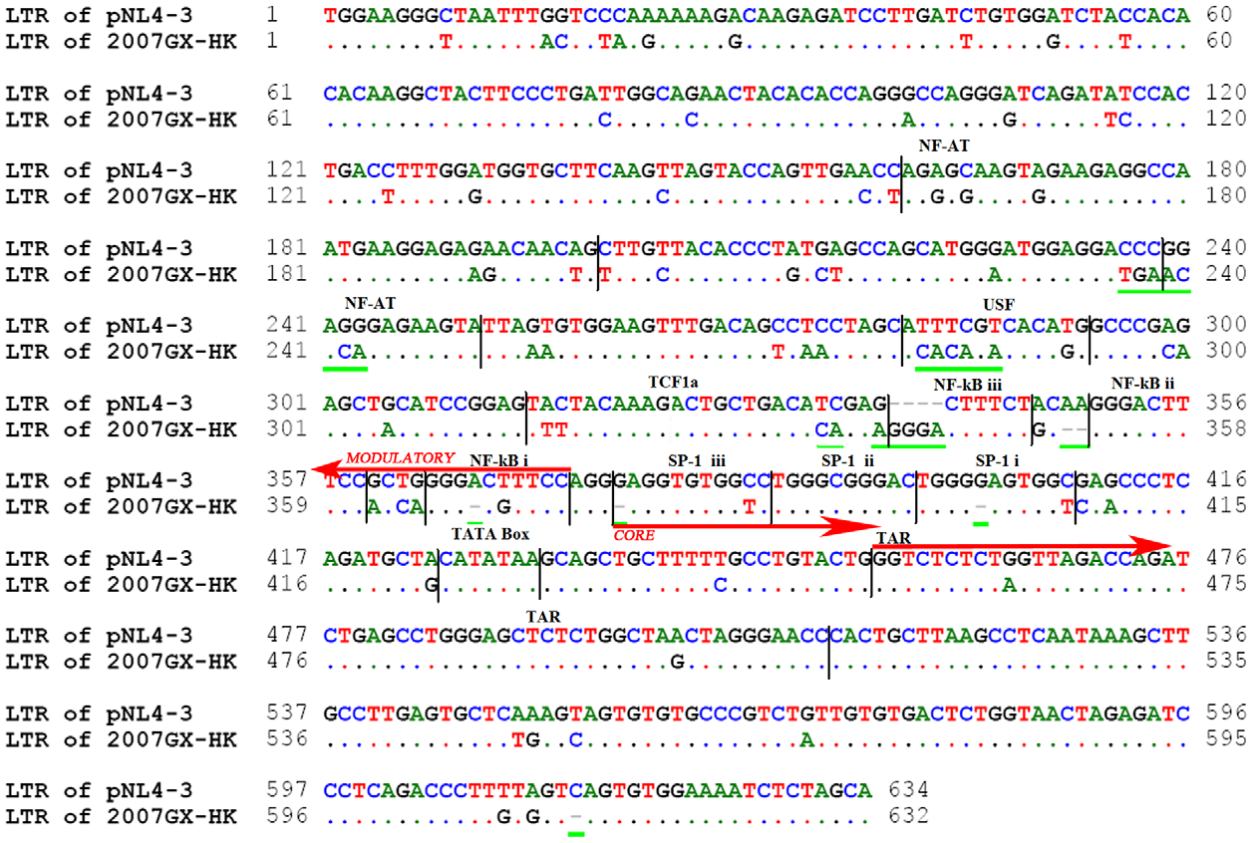
Alignment of the LTR sequences from pNL4-3 and 2007CNGX-HK. Both LTRs from pNL4-3 and 2007CNGX-HK were sequenced and their lengths were noted to be similar, at 634 bp and 632 bp respectively. However, there were seventy-eight base substitutions, six base deletions and four insertions observed between these two LTRs. There is a potential third NF-κB binding site (NF-κB iii) in the parental (2007CNGX-HK) LTR region due to the four base insertions at nt 340–343. Three single base deletions are also found amongst the NF-κB i, SP-1 iii and SP-1 i binding sites, respectively. The three major regulatory regions of LTR are indicated by vertical bars: Modulatory (NF-AT, USF, TCF1α, NF-κB), Core (SP-1, TATA box) and TAR (trans-activation region) elements. The green underlined sequences indicate selected base substitutions, insertions and deletions. Sequence identity is indicated by dots; insertions/deletions are indicated by dashes.

After transfecting the proviral molecular clones pBRNG and pBRGX into 293FT cells, p24 levels in the culture supernatant were detected, indicating replication-competence. However, the p24 level in the pBRNG-transfected culture supernatant was ∼4000-fold lower than that from the pBRGX-transfected culture (Fig. 2A). In addition, the X-Gal Staining assay revealed little infectivity in the pBRNG-transfected TZM-bl cells (data not shown). These results suggested that pBRGX may be an infectious clone. To confirm further whether the pBRNG and pBRGX-transfected cells could produce infectious viruses, PHA-stimulated PBMCs were infected with the supernatants from pBRNG and pBRGX-transfected cells respectively. The results showed that the viruses produced from pBRGX-transfected 293FT cells could infect PBMCs and replicate efficiently in the cells, reaching high level of p24 (over 70 ng/ml) in the culture supernatant at day 13 post-infection; its kinetic curve was highly similar to that of its parental virus strain (*P*>0.07). In contrast, the pBRNG-derived supernatants from the 293FT cells did not replicate well in PBMCs, compared with its parental isolate (*P*<0.02) (Fig. 2B). The LTR sequences of both 2007CNGX-HK and pNL4-3 were also compared, for insights into the possible mechanisms of replication-competence for pBRGX and pNL4-3 and for contrasting replication-incompetence for pBRNG (Fig. 3). The LTR lengths of pNL4-3 (subtype B) and parental 2007CNGX-HK (Subtype CRF_08BC) were similar, at 634 bp and 632 bp, respectively. Compared to LTR of subtype B, however, seventy-eight base substitutions, six base deletions and four base insertions were observed in parental LTR. After detailed analysis, it was noted that three single base deletions are amongst the NF-κB i, SP-1 iii and SP-1 i binding sites, respectively. Interestingly, compared to the subtype B's (pNL4-3) LTR, there is a potential third NF-κB binding site in the parental LTR region due to the 4 base insertions at nt 340–343, here named NF-κB iii. Other substitutions within NF-AT, USF, TCF1α and TAR elements were also evident (Fig. 3).

### The viruses derived from the infectious clone pBRGX and its parental primary isolate have CCR5 tropism

**Figure 4:**
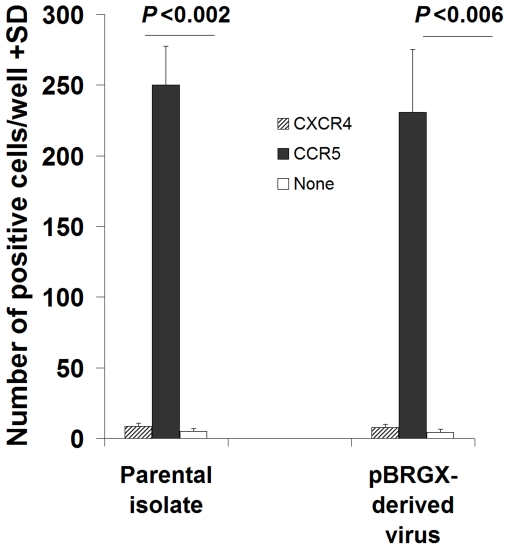
Co-receptor-mediated tropism of the pBRGX construct and its parental primary isolate. U373-MAGI-CXCR4cem (CXCR4), U373-MAGI-CCR5e (CCR5) and U373MAGI (none) cells were infected with either pBRGX-derived virus or its parental primary isolate. The infectivity of the virus was tested by the X-Gal Staining assay. The number of positives were scored per well and indicated along the y-axis.

To determine and confirm co-receptor-mediated tropism of these constructs, the infectivity of these pBRGX-derived viruses, and its primary isolate, was measured in U373-MAGI-CXCR4cem, U373-MAGI-CCR5e and U373MAGI cells using the X-Gal Staining assay. The results showed that while both the pBRGX-derived virus and its parental isolate could efficiently infect and replicate in U373-MAGI-CCR5e cells, they propagated in U373-MAGI-CXCR4cem and U373MAGI cells at a significantly lower level (*P*<0.01). This was at least 30-fold lower than that observed in the cells expressing CCR5 (Fig. 4). The results indicated and confirmed that both the infectious clone pBRGX-derived viruses and its parental primary isolate contain tropism to CCR5.

### Genomic DNA sequences and subtype identification

The full-length proviral genome of the HIV-1 CRF08_BC parental primary isolate was 9681 bp in size, spanning all structural and regulatory genes, and containing intact open reading frames (ORFs). No significant deletions, insertions or rearrangements were found in the proviral genome of the infectious molecular clone pBRGX. Detailed analysis of pBRGX revealed 72 mutations, relative to the parental primary isolate 2007CNGX-HK. Synonymous and nonsynonymous substitutions in the individual coding proteins of this infectious clone are listed in Table 1. All coding proteins, except Tat and Vpr, had synonymous substitutions; in contrast, Pol, Tat, Rev, Env and Nef proteins had non-synonymous substitutions, with an especially higher number of non-synonymous substitutions noted for Env (total 31), Rev (total 9) and Tat (total 7). The Pol and Nef proteins also had one and three nonsynonymous substitutions each. The effects of such nonsynonymous substitutions may be associated with the relative lower levels of replication observed for the pBRGX-derived viruses. This needs additional investigation for elucidation and confirmation.

**Table 1:** Base substitution mutations in the HIV-1 infectious clone pBRGX coding proteins as compared with the parental primary isolate 2007CNGX-HK.

Coding proteins	Substitutions	
	Synonymous	Non-synonymous
LTR	A47G, C160T, C300A (nt)	-
Gag	L-L, P-P, T-T, P-P	-
Pol	A-A, K-K, S-S	T-A
Vif	N-N, P-P	
Vpr	G-G	
Tat	-	P-S, Q-P, R-Q, Q-G, P-Q, T-P, G-E
Rev	Y-Y, Q-Q, Q-Q, R-R, R-R, V-V, L-L	K-E, G-R, R-N, A-K,C-S, A-T, A-T, S-T, S-P
Vpu	-	-
Env	G-G, L-L, Q-Q, K-K, L-L, R-R, L-L, L-L, P-P, P-P, S-S, R-R, A-A, L-L, S-S	R-K, S-N, M-E, Y-S, N-K, P-S, R-K, D-G, T-I, G-E, E-G, L-I, N-D, S-R, N-Y, K-R, T-S, T-Q, E-D, S-N, L-S, T-I, E-G, G-E, D-T, F-L, C-Y, V-L, V-G, S-N, K-R
Nef	L-L, P-P, L-L, C-C	R-Q, L-F, E-K
LTR	A9096G, G9349A, C9378T (nt)	-

**Figure 5:**
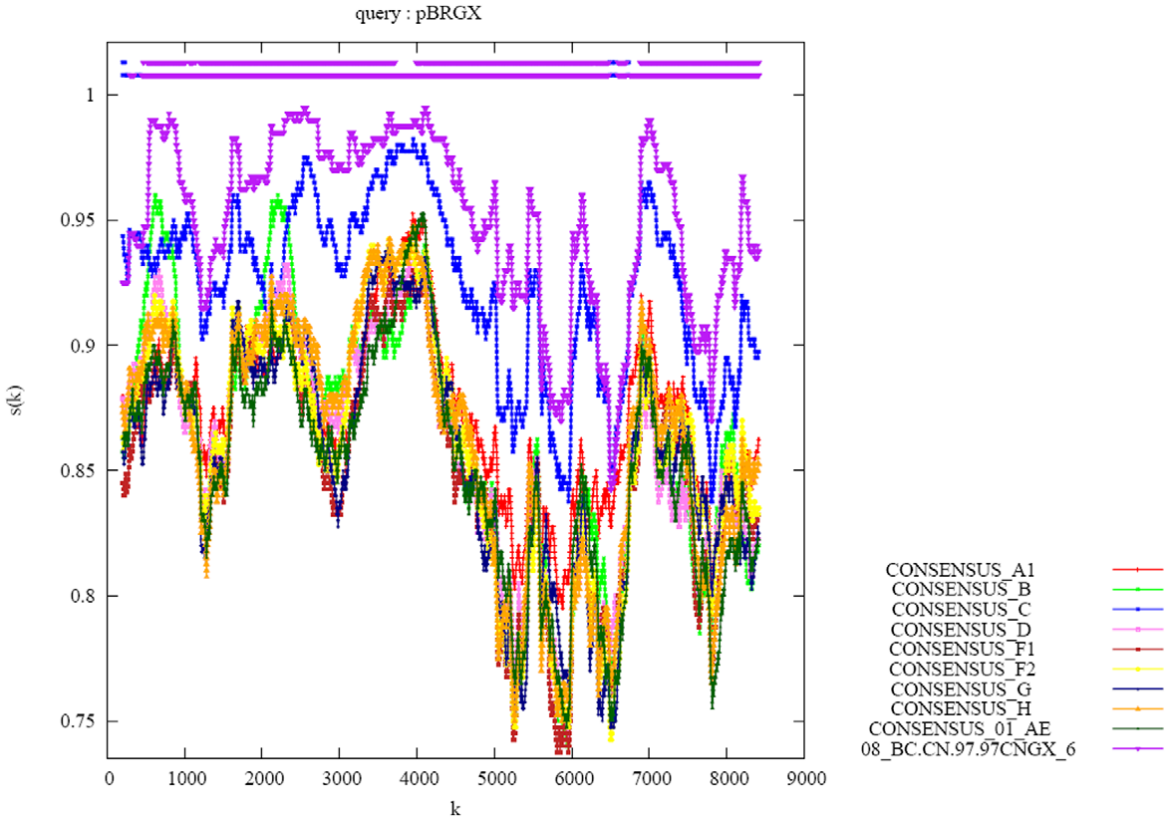
Similarity plots for the pBRGX strain. Genome recombination analysis of the pBRGX strain versus the reference subtype strains obtained from the Los Alamos database (http://www.hiv.lanl.gov/content/index), including Consensus_A1, B, C, D, F1, F2, G, H, 01AE and CRF08_BC.97CNGX_6F, are presented. SimPlot analysis was performed with web-accessible software, RIP 3.0 (http://www.hiv.lanl.gov/content/sequence/RIP/RIP.html), using a window size of 400 bp and a step size of 50 bp. As per the software protocol, the alignment was gap-stripped before analysis.

**Figure 6:**
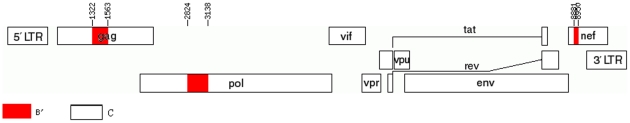
Schematic representation of the recombinant clone of pBRGX. The map of the construct pBRGX was generated using the Recombinant HIV-1 Drawing Tool, available from the LANL site (http://www.hiv.lanl.gov/content/sequence/DRAW_CRF/recom_mapper.html). Subtype B′ sequences are shown in red and subtype C sequences are displayed in white. The positions of six breakpoints are expressed as HXB2 coordinates at nt 1322, nt 1563, nt 2824, nt 3138, nt 8881 and nt 8950, respectively.

**Figure 7:**
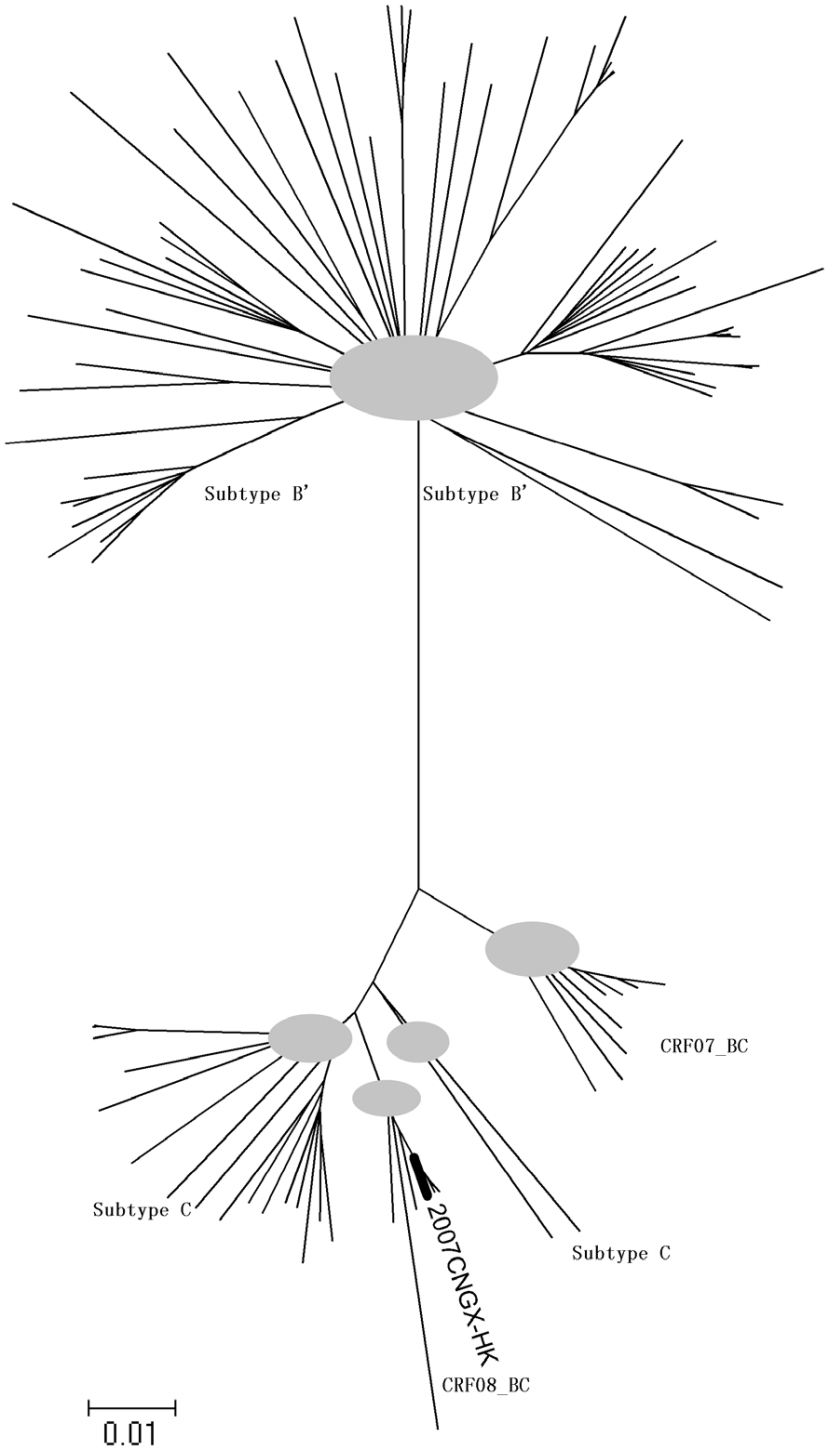
Phylogenetic reconstruction of HIV-1 subtypes and recombinants of Asian origin. A phylogenetic tree was generated from sequence alignments, and plotted using a neighbor-joining method. This was analysis implemented in MEGA 5 [35], [36] using 1000 bootstrap replicates. The optimum tree, with the sum of branch length equal to 2.05356961 is displayed. The evolutionary distances were computed using the Maximum Composite Likelihood method, available as a public software tool (http://www.megasoftware.net/WebHelp/part_iv___evolutionary_analysis/computing_evolutionary_distances/distance_models/nucleotide_substitution_models/hc_mcl.htm). The analysis included 113 nucleotide sequences *in toto*. Reference HIV-1 genomic sequences of subtypes B, C, 07BC and 08BC were used, and are available from the Los Alamos National Library (LANL). All positions containing gaps and missing data were eliminated (gap-stripped) as per the software algorithm. The 2007CNGX-HK genome formed a clade with the other CRF_08BC isolates. The grey circles indicate the junctions of the branches having close phylogenetic relationships.

Genome sequence recombination analysis using RIP3.0 demonstrated that this strain was a B′C recombinant, which is consistent with other CRF_08BC reference sequences (Fig. 5), in which two short fragments of subtype B′ were present in the gag and pol regions while the rest of the genome was closely related to subtype C (Fig. 6). Phylogenetic analysis of the full-length genome also revealed that this isolate clustered closely in a subclade with the other CRF_08BC reference sequences (Fig. 7). The proviral genomic sequences of 2007CNGX-HK and pBRGX reported in this paper are deposited in GenBank (accession numbers JF719819 and JF719818, respectively).

## Discussion

Since the first infectious clone, pNL4-3, was constructed in 1986, infectious clones for all subtypes of HIV-1, except CRF08_BC, have been constructed and widely used in the antiviral drug screening and HIV vaccine research fields [15]. As HIV-1 CRF08_BC is one of the major prevalent HIV subtypes circulating in China in recent years [7]–[9], [38], an infectious molecular clone was constructed and characterized for further biological studies of this HIV subtype and to aid in extending biotechnological applications, including vaccine development.

To date, almost all HIV infectious clones were constructed using very high-copy number vectors such as those from the pUC series [16], [21], [22], [39]. In this present study, the first molecular clone pNGX5, comprising the pNL4-3 vector and the 2007CNGX-HK genome, except for its LTRs, was constructed using a similar strategy. As discussed, and in contrast to pNL4-3, this initial clone was highly unstable in host bacterial cells during the culturing and plasmid amplification processes. After two generations, smaller sized recombinant plasmids predominated. In retrospect, this is not unusual because it has been reported that homologous recombination between LTR regions is frequently observed during molecular cloning and amplification in *E. coli* cultures, even if using *Rec*A (-) bacterial strains as hosts [40]. However, it is still not entirely clear why pNL4-3 is stable in contrast to the unstable clone which was based on a pNL4-3 vector. One possible explanation may be that the very high-copy number origin of replication (ori) of pNL4-3 is not compatible with the large genome of 2007CNGX-HK. In a previous study, a full-length infectious clone of human adenovirus type 3 was constructed successfully using pBR322, a moderate-copy number plasmid. This adenoviral insert was ∼40 kb long, but was found to be stable in *E. coli* hosts [41]. Therefore, it was suggested that a moderate-copy number plasmid might be more compatible with a large HIV genome insertion. To test this hypothesis, the proviral genome of pNGX5 was subcloned into pBR322 to generate the second molecular clone pBRNG. This construct harbored LTRs from pNL4-3 along with a majority of the genome of 2007CNGX-HK, i.e., missing its LTR. A third plasmid pBRGX harboring the full-length genome of 2007CNGX-HK was also constructed, by replacing the LTRs of pNL4-3 in pBRNG with the equivalent parental LTRs from 2007CNGX-HK. These two clones were very stable during subsequent amplification in *E. coli* cultures, even after 20 passages of the cultures. This confirmed the hypothesis.

In subsequent experiments, it was observed that pBRNG did not efficiently replicate or produce infectious virus (Fig. 2). Meng *et al*, reported earlier of the construction of an infectious clone of CRF07_BC, i.e., NLXJDC6441X2 [16]. Similar to the pBRNG construct reported here, NLXJDC6441X2 contains both of the pNL4-3 LTRs along with the majority of the genome of CRF07_BC, that is, except for the CRF07_BC LTRs. However, this construct also produced a lower replication yield than expected [16]. These observations suggested that the LTRs of both 2007CNGX-HK and NLXJDC6441X2 may play an important, if not essential, role in the production of infectious virus particles. In our study, when the LTR of B subtype in PBRNG was replaced by parental LTR in PBRGX, the later clone produced infectious viruses which were able to replicate in PBMCs efficiently. Viral replication kinetics of this pBRGX-derived virus was highly similar to its parental isolate. The results suggested that the parental LTRs may be important for the construction of replication-efficient infectious clones, at least for some HIV subtypes such as CRF_BC.

The LTR of HIV-1, which functions presumably as a promoter, can be divided into three major regulatory regions, i.e., modulatory, core and TAR elements. It was reported that the modulatory element is critical in altering viral gene expression in response to changes in cellular signal transduction pathways [42]–[44]. The core element is critical for both basal and tat-induced gene expression, and the cellular factors binding to this region are noted to influence the associations with general initiation factors, which are important in activating HIV-1 gene expression [45]–[47]. TAR element is critical for tat activation of HIV-1 LTR in all human cell lines tested [48]–[50]. Successful HIV replication includes important post-entry events, including viral transcription, replication and viral turnover, during which the regulatory regions of HIV-1, especially the LTRs, function as promoters/enhancers to enable the HIV-1 genome to replicate efficiently through differential recruitment of cellular transcription factors [51], [52]. By compared the sequences of parental (2007CNGX-HK) and subtype B's (pNL4-3) LTRs, we found 19, 2 and 2 base substitutions in modulatory, core and TAR elements, respectively (Fig. 3). Interestingly, the four base insertions at nt 340–343 resulted in a potential third NF-κB (iii) binding site in parental LTR (Fig. 3). Presumably, three NF-κB binding sites may be more efficient than two for initialing the viral replication in CRF08_BC subtype. Although only a single base deletion (A or G) was found respectively in NF-κB i, SP-1 iii and SP-1 i binding regions of parental LTR, this subtle difference within the LTR promoter might have a significant impact upon viral replication kinetics. For example, it has been reported that the higher transmission efficiency of CRF01_AE, versus the North American subtype B, is due to a single nucleotide deletion in one of the upstream NF-κB binding sites of the CRF01_AE LTR. This mutation apparently creates a binding site for the GABPα and GABPβ1 transcription factors [53]. Each HIV-1 subtype has a specific and peculiar LTR promoter configuration, and apparently even minor sequence changes in the transcription factor binding sites (TFBs) or their arrangements can impact transcriptional activity [54]. De Arellano *et al*. also found that a G to A hypermutation at presumably critical TFBs within the LTRs compromised the activity of the viral promoter [55]. However, whether and how these base substitutions, deletions and insertions identified in the parental LTRs could influence the viral promoter activity are still unclear yet, which need to be further investigated.

Similar to the CRF07_BC infectious clone NLXJDC6441X2-derived virus and its parental isolate [16], the co-receptor-mediated analysis demonstrated that both the pBRGX-derived virus and its parental isolate, CRF08_BC, contain tropism to CCR5. Notably, the viruses generated from the CRF08_BC infectious clone pBRGX exhibited higher titers after culturing in PBMCs, when compared with the reported CRF07_BC infectious clone NLXJDC6441X2-derived virus, which showed poor replication ability [16]. According to Meng, *et al*., the replication dynamics curve suggested that NLXJDC6441X2 is most likely replication-incompetent because of its lower p24 levels (less than 100 pg/ml) and the lack of p24 variation in PBMC culture [16]. It has been reported that the LTRs may function as “sticky ends”, which are recognition and binding sites for the integrase protein, as well as function as promoters/enhancers to drive the expression of the genome [56]–[58]. The difference between the viral titers of these two infectious clone-derived viruses may be most likely attributed to their replication ability but not infectivity. An intriguing conjecture is that the LTRs of NLXJDC6441X2 may also play an important role in the production of infectious virus particles. Further investigations are warranted, for example, the construction of an infectious clone containing the full-length of CRF07_BC by replacing the LTRs of NLXJDC6441X2 with the parental LTR of original CRF07_BC; comparison of the differences between LTRs sequences of NLXJDC6441X2 versus its parental isolate; and subsequent identification of potential key motif(s) contained within the LTRs by site mutation assays. These are needed for understanding further the biological and essential functions and roles of the LTRs in virus replication, particularly as they relate to HIV biology and the on-going biotechnological efforts to develop vaccines.

The phylogenetic analysis and tree visualization showed the 2007CNGX-HK subtype forming a subclade with other CRF08_BC strains, and was more closely related to subtype C than to subtypes B′ and CRF07_BC. This result was consistent with the genome recombination analysis (BootScan plot) that showed short fragments of the subtype B sequences inserted into the subtype C strain genome, at the gag and pol locations. Interestingly, two other isolates, C.IN.2003.D24 and C.IN.1994.94IN476, which were previously identified as subtype C in the Los Alamos database, are more closely related to CRF08_BC rather than to subtype C, as indicated from the phylogenetic tree generated (Fig. 7). This, too, calls for further analysis to confirm the true subtypes of these two strains.

This report provides data and observations that demonstrate the parental LTRs are important for generating an efficient, replication-competent, infectious molecular clone of CRF08_BC. This observation may be useful in the generation and development of infectious clones from other HIV subtypes, as is the observation of a preference for a moderate-copy number vector. The availability of this particular replication-competent infectious clone will enable systematic investigations of CRF08_BC *in vitro*, including its use as a tool in continuing research on antiviral drug susceptibility and resistance. This clone will also facilitate the development of effective vaccine candidates in the near future, especially as drug resistance becomes more wide-spread and a major issue. For example, the NRTI resistance mutation T69S has been reported in 94% (30/32) of the CRF_08BC subtypes among IDUs in the Guangdong Province of China [59]. The availability of this infectious clone may also help in developing a new antiviral drug for CRF_08BC, which, again, has become a predominant HIV subtype circulating in China.
